# Development of nebulized inhalation delivery for fusion-inhibitory lipopeptides to protect non-human primates against Nipah-Bangladesh infection

**DOI:** 10.1016/j.antiviral.2025.106095

**Published:** 2025-01-25

**Authors:** Olivier Reynard, Mathieu Iampietro, Claire Dumont, Sandrine Le Guellec, Stephanie Durand, Marie Moroso, Elise Brisebard, Kévin P. Dhondt, Rodolphe Pelissier, Cyrille Mathieu, Maria Cabrera, Deborah Le Pennec, Lucia Amurri, Christopher Alabi, Sylvain Cardinaud, Matteo Porotto, Anne Moscona, Laurent Vecellio, Branka Horvat

**Affiliations:** a CIRI, Centre International de Recherche en Infectiologie, INSERM U1111, CNRS, UMR5308, Univ Lyon, Université Claude Bernard Lyon 1, École Normale Supérieure de Lyon, 21 Avenue Tony Garnier, 69007, Lyon, France; b DTF-Aerodrug, R&D Aerosoltherapy Department of DTF Medical (Saint Etienne, France), Faculté de Médecine, Université de Tours, 37032, Tours, France; c INSERM, P4 Jean Mérieux, 69007, Lyon, France; d UMR703, PAnTher APEX, INRAE/Oniris, Nantes, Frnce; e Division of Pediatric Critical Care Medicine and Hospital Medicine, Department of Pediatrics, Vagelos College of Physicians and Surgeons, Columbia University Irving Medical Center, New York, USA; f CEPR, INSERM U1100, Université de Tours, Tours, France; g Robert Frederick Smith School of Chemical and Biomolecular Engineering, Cornell University, Ithaca, NY, USA; h Vaccine Research Institute, Créteil, France; i Inserm U955, Team 16, Institut Mondor de Recherche Biomédicale, Université Paris-Est Créteil, Créteil, France; j Center for Host-Pathogen Interaction, Vagelos College of Physicians and Surgeons, Columbia University Irving Medical Center, New York, USA; k Department of Microbiology & Immunology and Department of Physiology & Cellular Biophysics, Vagelos College of Physicians and Surgeons, Columbia University Irving Medical Center, New York, USA; l Department of Experimental Medicine, University of Campania Luigi Vanvitelli, Caserta, Italy

**Keywords:** Nipah virus, Nebulization, Virus-cell-fusion, Inhibitory lipopeptides, Non-human primates, Antivirals

## Abstract

Nipah virus (NiV) is a lethal zoonotic paramyxovirus that can be transmitted from person to person through the respiratory route. There are currently no licensed vaccines or therapeutics. A lipopeptide-based fusion inhibitor was developed and previously evaluated for efficacy against the NiV-Malaysia strain. Intraperitoneal administration in hamsters showed superb prophylactic activity and promising efficacy, however the intratracheal delivery mode in non-human primates proved intractable and spurred the development of an aerosolized delivery route that could be clinically applicable. We developed an aerosol delivery system in an artificial respiratory 3D model and optimized the combinations of flow rate and particle size for lung deposition. We characterized the nebulizer device and assessed the safety of lipopeptide nebulization in an African green monkey model that mimics human NiV infection. Three nebulized doses of fusion-inhibitory lipopeptide were administered every 24 h, resulting in peptide deposition across multiple regions of both lungs without causing toxicity or adverse hematological and biochemical effects. In peptide-treated monkeys challenged with a lethal dose of NiV-Bangladesh, animals retained robust levels of T and B-lymphocytes in the blood, infection-induced lethality was significantly delayed, and 2 out of 5 monkeys were protected from NiV infection. The present study establishes the safety and feasibility of the nebulizer delivery method for AGM studies. Future studies will compare delivery methods using next-generation fusion-inhibitory anti-NiV lipopeptides to evaluate the potential role of this aerosol delivery approach in achieving a rapid antiviral response.

## 1. Introduction

Nipah virus (NiV) is a zoonotic paramyxovirus, a member of the *Henipavirus* genus, that causes severe respiratory and neurological disease with case-fatality rates ranging from 40% to 100% in documented outbreaks (Moore et al., 2024). Widely distributed *Pteropus* bats are a natural reservoir of NiV in South-East Asia, posing a constant threat of spillovers (Enchéry and Horvat, 2017). Propensity for NiV respiratory disease seen in India and Bangladesh and frequent human-to-human transmission has raised the concern of pandemic potential (Luby, 2013) and led to the designation of NiV infection among the priority diseases for epidemic preparedness by WHO as a part of its Blueprint list for Action to prevent Epidemics (Ukoaka et al., 2024). Two major NiV strains have been identified so far, NiV-Malaysia and NiV-Bangladesh, that induce different symptoms in patients, with the currently circulating Bangladesh strain being more pathogenic and causing a more severe pulmonary syndrome (Soman Pillai et al., 2020). To date, no licensed therapeutic drugs or vaccines are available to combat NiV disease in humans.

NiV infects a broad range of cell types including endothelial cells, epithelial cells and neurons (Pelissier et al., 2019). After initial binding to the cell surface co-receptor heparan sulfate (Mathieu et al., 2015), the NiV attachment protein G interacts with ephrin B2 and/or B3 cell receptors, triggering conformational changes in the NiV fusion protein F, exposure of the viral fusion peptide, and fusion between the virus and host cell membrane coincident with formation of a 6-helix bundle between the C- and N-terminal repeats of F (White et al., 2023). We have shown that peptides derived from the C-terminal heptad repeat regions (HRC) of paramyxovirus F proteins interfere with the initial steps of viral entry by blocking formation of the F’s 6-helix bundle structure (Outlaw et al., 2021; Porotto et al., 2006, 2007). NiV-mediated membrane fusion and viral entry can be potently inhibited using lipid-conjugated peptides derived from the C-terminal heptad repeat of the human parainfluenza type 3 virus (hPIV3) F protein (Porotto et al., 2006, 2007, 2010a, 2010b).

NiV-Malaysia infection can be entirely prevented with fusion-inhibitory lipopeptides derived from the HPIV3 HRC delivered parenterally in Syrian hamsters and treatment even several days into infection effectively reduced lethality (Porotto et al., 2010a). On this foundation, efforts were undertaken to administer the NiV inhibitory peptides by a a respiratory route. In Syrian hamsters infected with a very high dose (10^6^ PFU) of NiV-Malaysia, intranasal (i.n.) peptide dosing for 3 days at the start of infection resulted in approximately 50% survival (Mathieu et al., 2018), suggesting that i.n. peptide delivery was insufficient to completely block infection. Attempted dosing of non-human primates (NHP) with peptide given intratracheally (i.t.) led to expectoration of peptide on the first day of treatment in most of the treated animals (Mathieu et al., 2018), providing no clarity about whether airway delivery was effective in NHP. The NHP were infected with a high dose of NiV (2 × 10^7^ PFU), providing a remarkably high bar for antiviral efficacy, and yet in the face of the expectorated peptide doses survival was 33%, a figure that clearly underestimated the potential of the peptide antiviral treatment. These technical issues spurred the development here of an aerosolized delivery route for NiV treatment that would circumvent the problems we faced in i.n. and i.t. delivery, for assessing the potential for airway delivery as a component of NiV antiviral treatment.

We recently showed that aerosolization of fusion-inhibitory lipopeptide was suitable for preventing infection by measles virus, another paramyxovirus, in cynomolgus monkeys (Reynard et al., 2022). In the case of measles, different to NiV, aerosolized administration is sufficient to block the infection. Therefore, to advance with the development of a respiratory route delivery approach for NiV antiviral peptides that could be clinically applicable, we characterized a nebulizer prototype able to deliver lipopeptides to the airway in the African green monkey (AGM) model. We demonstrate the feasibility and safety of aerosolizing fusion-inhibitory lipopeptides using a nebulizer adapted to the NHP respiratory system, and we test the mesh nebulizer and face mask system in the AGM. This study did not compare nebulizer delivery with previous delivery approaches and, therefore, does not determine the optimal delivery route for antiviral efficacy. However, it demonstrates the feasibility of using nebulizer delivery for NiV antiviral lipopeptides in AGMs for future research.

## 2. Materials and methods

### 2.1. Peptide synthesis

The fusion-inhibitory peptide VIKI-chol-PEG4 (“VIKI”) (Porotto et al., 2010a) contains 36 amino-acids from C-terminal Heptad Repeat (HRC) domain of the human parainfluenza virus 3 (HPIV3) fusion protein, and a small flexible linker (Fig. 2A), with mutations in the F protein: E459V, A463I, Q479K, K480I, as described previously (Porotto et al., 2010a). Unconjugated peptide (Shanghai Ruifu Chemical Co.) was coupled to a four-mer polyethylene glycol (PEG) linker and bromoacetyl cholesterol (Charnwood Molecular, Ltd) as previously described (Ingallinella et al., 2009; Porotto et al., 2010a). For nebulization prior to NHP administration, VIKI peptide was initially dissolved in 1% DMSO/water milliQ to obtain a final concentration of 4 mg/ml (pH 7.0 adjusted with NaOH and stabilized with 10 mM HEPES).

### 2.2. Cells and virus

VeroE6 and HEK 293T cells were grown in DMEM glutamax (ThermoFisher Scientific, 61965026) supplemented with 10% fetal bovine serum (FBS, Biowest ref. 91830–500), glutamine and antibiotics (100U/mL of penicillin and 100 μg/mL of streptomycin) in 5% CO_2_ 37°C incubators. NiV Bangladesh (NiV) isolate SPB200401066 (CDC, Atlanta, USA; GenBank AY988601) was produced at the INSERM BSL4 Jean Mérieux and titrated by plaque assay on VeroE6 cells. Both cell lines and viral stocks tested negative for mycoplasma (MycoAlert, Lonza).

### 2.3. Fusion inhibition and viral plaque reduction assay

HEK 293T cells transfected with the omega reporter subunit of β-gal (“target cells”) were incubated with cells co-expressing viral glycoproteins (Nipah G and F) and the alpha reporter subunit of β-gal (“effector cells”) in the absence or presence of inhibitory peptides. The β-gal activity was analyzed using the luminescence-based kit, Galacto-Star β-galactosidase reporter gene (ThermoFisher), as described (Mathieu et al., 2018). The inhibitory effect was calculated as the ratio of the relative luminescence units in the presence of different concentrations of peptide, corrected for background luminescence. For viral plaque reduction assays, Vero cells were plated in 6-well plates and 24h later exposed to several dilutions of VIKI lipopeptide for 1h. The treatment was removed and cells were infected with NiV (50 PFU). After 1h the inoculum was removed and the cells were covered with 1.2% carboxymethylcellulose in DMEM 3%FBS, incubated for 3 days at 37 °C and then stained with crystal violet. Viral plaques were counted and plaque reduction was analyzed using Prism software. IC_50_ was calculated using nonlinear fitting, and [antagonist] versus normalized response-variable slope parameter.

### 2.4. Cell toxicity assay

Vero cells were incubated for 96h with VIKI lipopeptide at the indicated concentrations up to 5 μM. VIKI lipopeptide was added to the media at 37 °C and viability was determined after 24h using the Vybrant MTT (3-(4,5-dimethylthiazolyl-2)-25-diphenyltetrazolium bromide) cell proliferation assay kit (ThermoFisher Scientific), according to the manufacturer’s guidelines. TritonX-100 (1%) was used as a positive control. Absorbance was read at 540 nm using a Tecan M1000PRO microplate reader.

### 2.5. Viral load quantification by RT-qPCR

Viral RNA was extracted using Qiamp Viral RNA Kit (Qiagen) for swab samples and RNeasy Mini-Kit (QIAGEN) for organs. Viral load was evaluated by one-step RT-qPCR (NEB Luna^®^ Universal One-Step RT-qPCR kit) using NiV-N-specific primers(NiV-N FW: 5′-GTGCTGAGTAT ACCCCACC-3′ and NiV-N Rev: 5′-GAGATAAGCGCGGACAAGA-3′) and GAPDH primers (GAPDH FW: 5′-CACCCACTCCTCCACCTTTGAC-3′, GAPDH REV: 5′-GTCCACCACCCTGTTGCTGTAG-3′). PCR amplification was recorded on a StepOne plus device (ThermoFisher Scientific). All samples were run in duplicates, and results were analyzed using ABI StepOne software v2.1 (Applied Biosystems).

### 2.6. Nebulizer device

A customized nebulizer and facial mask, specifically designed for NHPs (Vecellio Laurent, 2023), was used to administer the VIKI lipopeptide to AGMs via aerosolization. The static mesh nebulizer prototype (DTF medical, Saint-Etienne, France) includes a 3 μm hole mesh and a piezo-electric system. The facial mask was connected to an absolute filter to prevent ambient air contamination during animal respiration. According to previous studies (Cabrera et al., 2023; Reynard et al., 2022), this prototype nebulizer can deliver a high amount of aerosol to NHP’s lungs in a short inhalation time (approximately 10min). The particle size was previously determined to have a mean volume diameter between 4.1 and 4.7 μm, a respirable fraction (particles smaller than 5 μm) between 53% and 61%, and a flow rate between 0.3 mL/min- 0.4 mL/min (Reynard et al., 2022).

### 2.7. Measurement of aerosol characteristics

Peptides were nebulized and collected in a collection cup at the outlet of the mesh nebulizer. Fractions before and after nebulization were compared in fusion and viral inhibition assays. Lipopeptide aerosol flow rate was measured by weighing the nebulizer before and after nebulization to assess the volume nebulized over a defined time. The 27 nebulizer devices used on animals during the study had an average particle size diameter of 4.2 μm ± 0.3 μm, and an aerosol flow rate of 0.47 mL/min ±0.08 ml/min.

### 2.8. Aerosol deposition prediction using a 3D NHP cast-printed model

**Fig. 1.**
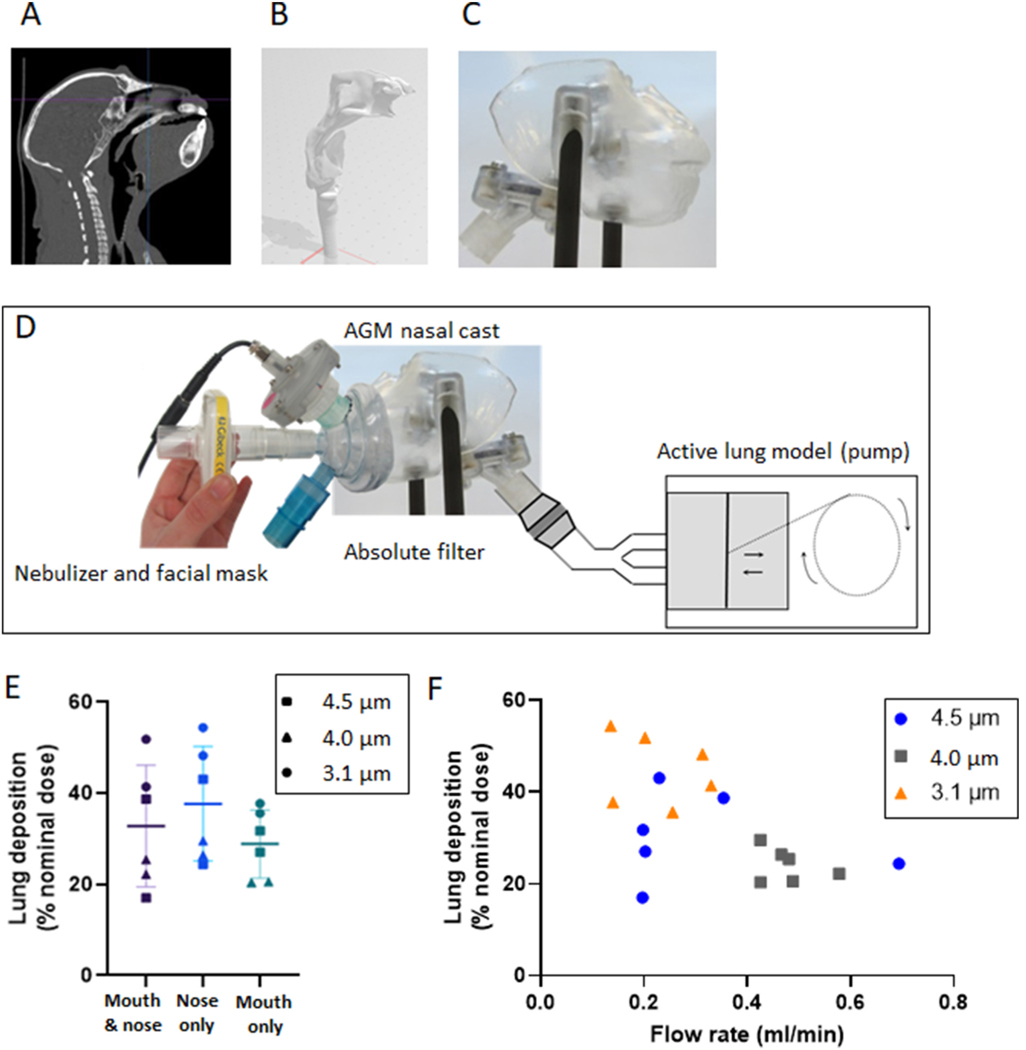
Experimental set up for the measurement of aerosol deposition using a 3D print cast AGM model. (A) Computed tomography (CT-Scan) of AGM upper airway sagittal view, (B) (C) Numerical 3D cast model from CT-scan; (D) Experimental set up of 3D print AGM cast, with respiratory pump, 3D cast and nebulizer with face mask. (E) Prediction of lung deposition using a breathing demonstrator, considering different breathing modes and aerosol particle sizes. (F) Influence of flow rate and particles sizes on lung deposition analyzed using the breathing demonstrator.

A 3D resin model of an entire head of AGM, including both nasal and buccal cavities, was created using stereolithography from raw computed tomography CT-Scan of AGM (Fig. 1). The model was then connected to an active lung model reproducing AGM respiratory function and an absolute filter was used to collect aerosols that reached the lower airways model. The study employed specific AGM breathing parameters: a tidal volume of 25 mL, a respiratory rate of 35 cycles/min, and an I/E ratio of 1/1, as previously reported (Casacó et al., 2010; Foster et al., 2008). To consider breathing variability during anesthesia, different breathing modes of the AGM were simulated on the demonstrator including nose-only, mouth-only, and nose&mouth respiratory patterns. The nebulizer was loaded with 1 mL of 1% sodium fluoride. Electrochemical dosage was used to assess aerosol deposition in both filter and 3D cast (Dennis et al., 1990). The percentage of aerosol deposited was then calculated based on the nominal dose for lipopeptide.

### 2.9. Ethical approval for animal experimentation

Animal experiments involved 20 AGMs (*Chlorocebus sabaeus),* weighing from 2.2 to 3.9 kg, from Saint-Kitts, obtained by SARL-Bioprim, Baziège, France. Pharmacology and toxicology experiments were performed at Cynbiose primate facility (Marcy l ‘Etoile, France), and reviewed by the Animal Welfare Body of Cynbiose and the Ethics Committee of VetAgro-Sup and approved under number 1465V3 (MESR number: 2016072117544328). The LD_50_ and challenge experiments were performed at Inserm Jean Merieux BSL4 laboratory under ethical agreement (DAP-APAFIS# 13016_2018011216438287_v2, DAP-APAFIS #12649_20171215111485_v7 and APAFIS#37780–2022062310304143 v4).

### 2.10. Study of lipopeptide biodistribution and toxicology in AGM

To determine safety of the aerosolized VIKI lipopeptide in the NHP model, three adult AGMs were treated with nebulized peptide for less than 15min with VIKI lipopeptide (3 mL at 4 mg/ml) and one AGM with saline solution (mock). Blood samples were taken at 24, 48 and 72h post-treatment and blood biochemistry and hematology analysis were performed on a KONELAB KL30 ISEND (ThermoFisherScientific) and XT2000i Vet automatic analyzer (Sysmex) respectively. After 18 days, the same animals were subjected to nebulization for three consecutive days and the blood samples were taken 24h after the last nebulization.

### 2.11. NiV infection of AGMs

To determine the dose of the virus to use in experiments, ten young adult AGMs were divided into three groups and challenged via the intratracheal route with 10^2^ (n = 3), 10^4^ (n = 4) and 10^6^ PFU (n = 3) of NiV Bangladesh strain in 1 mL of DMEM, under anesthesia (IM, Zoletil^®^). Endotracheal probes were then flushed with 2 mL saline solution, resulting in a total inoculation volume of 3 mL. Animals were monitored daily and were provided with OWM banana diet (SDS Dietex), with regular enrichment, fruits, and water *ad libitum*. Weight, temperature, hematology (MS9 analyzer, MS Labo) and biochemistry (Pentra C200 analyzer, Horiba) parameters were evaluated under anesthesia (IM, Zoletil^®^) at day 0, 2, 4, 6, 8.

The NiV dose of 10^2^ PFU was selected for further peptide nebulization experiments, inducing 100% lethality. Seven AGMs were divided into two groups: control (two males) which received mock treatment (1% DMSO in saline solution), and lipopeptide treated group (two males and three females) 3 times (−24, −6, +24h post infection) with 3 mL VIKI lipopeptide at 4 mg/ml. Animals were monitored daily as described above and biochemistry parameters were evaluated under anesthesia on day −1, 2, 5, 7, 9, 12, 14, 19, 21, 23 post-infection (Pentra C200 analyzer, Horiba). The comparative analysis also included a historical cohort of untreated AGMs infected with 10^2^ PFU of NiV, two animals originating from the initial study to assess virus lethal doses and 3 animals from a previously performed vaccine trial [unvaccinated animals, not treated with nebulizer (Pastor et al., 2024)]. Clinical exams were performed daily post-challenge by scoring: temperature, weight, dehydration, breath, reactivity and neurological symptoms. Euthanasia was performed when a human endpoint was reached with the score superior to 15, or at the end of the study 27–28 days after infection, by injecting intracardially 5 mL of Euthasol. Necropsies were performed and organs were fixed using 4% formaldehyde (Merck) during two series of 7 days incubation and then processed for histopathological analysis.

### 2.12. Immunofluorescence and histology

To assess the bioavailability of VIKI peptide in lungs, 5 μm slices of paraffin embedded organs were stained and imaged by confocal microscopy as described previously (Welsch et al., 2013). Briefly, after being blocked and permeabilized in 0.1% TritonX100, 5% BSA solution, slices were sequentially incubated with a polyclonal rabbit anti-VIKI Ab (custom made, Genscript, Netherlands overnight at 4 °C and with a secondary goat anti-rabbit alexa-555 (Thermo) and DAPI for 1h at room temperature (RT). Slides were imaged using a Zeiss LSM800 confocal microscope Platim facility (Lyon, France). For histopathology, formalin-fixed specimens were embedded in paraffin wax, 4 μm tissue sections were processed and stained with haematoxylin-eosin-saffron (HES). For immunohistochemistry, the sections were dewaxed and rehydrated. Slides were incubated for 30min at RT with 5% FBS diluted in PBS, followed by incubation overnight at 4 °C with rabbit polyclonal anti-NiV-N antibody diluted in 5% FBS, 0.1% Tween20, PBS 1X solution. The slides were then rinsed and incubated for 30min at RT with secondary goat anti-rabbit IgG, Alexa Fluor 555 (1 μg/ml) and DAPI (Sigma, 0.1 μg/ml) diluted in 5% FBS, 0.1% Tween20, PBS 1X solution. Finally, the slides were mounted with Fluoromount G (Thermo). Images were acquired using a Nikon Eclipse TS2R microscope. The histopathologic evaluation was performed by a board-certified veterinary pathologist. Fluorescence intensity of DAPI and VIKI in lung slides of AGMs were determined using ImageJ software and graphically represented using GraphPad Prism.

### 2.13. Peripheral blood mononuclear cell phenotyping by cytoflyorometry

Blood (1 mL) was collected in EDTA tubes from each AGM and added to 10 mL of PharmLyse 1X (BD Biosciences) to eliminate red blood cells. Tubes were mixed by successive reversals and incubated at RT for 15min in the dark. Then, the mix was centrifuged at 200×*g* for 5min at RT, the supernatants were removed, and cell pellets were resuspended in 1 mL of PBS +1%FBS and washed once in PBS+1%FBS and then resuspended in 150 μL of PBS +1%FBS containing the cocktail of antibodies during 20min at 4 °C in the dark: anti-CD3-AF488 clone SP34–2 (BD, #557705), CD20-APCH7 clone 2H7 (BD, #560853) and CD14-APC clone m5e2 (BD, #555399). Then, cells were washed twice with 1 ml of PBS +1% FBS 5min at 1,500 rpm and fixed with 150 μL of formaldehyde 4% methanol-free for 15min at 4 °C in the dark. Finally, 1 mL of PBS was added and centrifuged at 2,500 rpm for 5min. Final pellets were resuspended in 500 μL of PBS, read in a Gallios flow cytometer (Beckman Coulter) and analyzed with Flowjo V.10.8 (Treestar).

### 2.14. Seroneutralization

Serial dilutions of sera were done in DMEM containing 2% FBS, mixed with 50 PFU of NiV-B, incubated 30min at 37 °C, and transfected into VeroE6 cells in 96 well-plates for 60min. DMEM with 10% FBS was added after 1h and the cells were incubated for 2 day at 37 °C. Cytotoxic effects were evaluated after crystal violet staining as described (Pastor et al., 2024).

### 2.15. Statistical analysis

Statistical analysis of the differences in the survival was performed using log-rank Mantel Cox test. Flow cytometry results are displayed using curves representing the mean of each cell population for each group and the area within error bands represent the standard deviation (SD) for n = 5 animals. Statistical significance was assessed by *t*-test, followed by Mann-Whitney test; *p < 0.05, **p < 0.01 using GraphPad Prism 8.3 software.

## 3. Results

### 3.1. Determination of aerosol deposition using a 3D print cast AGM model

In addition to particle size, aerosol flow rate has been recently identified to be a key parameter influencing the deposition of inhaled aerosols in the respiratory tract (Cabrera et al., 2023). However, even under controlled parameters the deposition of aerosols can be affected by the breathing mode of animals during inhalation (MacLoughlin et al., 2016). AGMs have been used as the most appropriate NHP model due to their high sensitivity to NiV infection (Geisbert et al., 2010). In order to model lung deposition in AGM, we reconstituted an AGM respiratory tract based on CT-scan imaging (Fig. 1A–C) and generated a 3D-printed demonstrator connected to a breathing machine (Fig. 1D). The 3D model was recently shown to be useful for predicting total aerosol deposition in a macaque model of NHP airways (Creppy et al., 2023). Breathing through the mouth only resulted in deposition of 29 ± 7%, compared to 38 ± 13% for nose-only breathing and 33 ± 13% for mouth and nose combined (Fig. 1E), suggesting that breathing modes do not significantly impact lung deposition. To comply with *in vivo* delivery requirements that ideally combine short delivery to minimize anesthesia and maximal deposition, we assessed lung deposition of several combinations of flow rate/particles size. We observed the most efficient lung deposition with a low flow rate combined with small aerosols (Fig. 1F), similar to what has been recently shown with the macaque model (Cabrera et al., 2023). To ensure the rapid delivery of the drug to animals with limited duration of anesthesia, we chose a flow rate of 0.5 ml/min and an aerosol size of 4 μm. Those results showed that regardless of the breathing mode, the nebulizer delivered at least 20% of the dose of the compound to the lung.

### 3.2. Characterization of nebulized lipopeptides

**Fig. 2.**
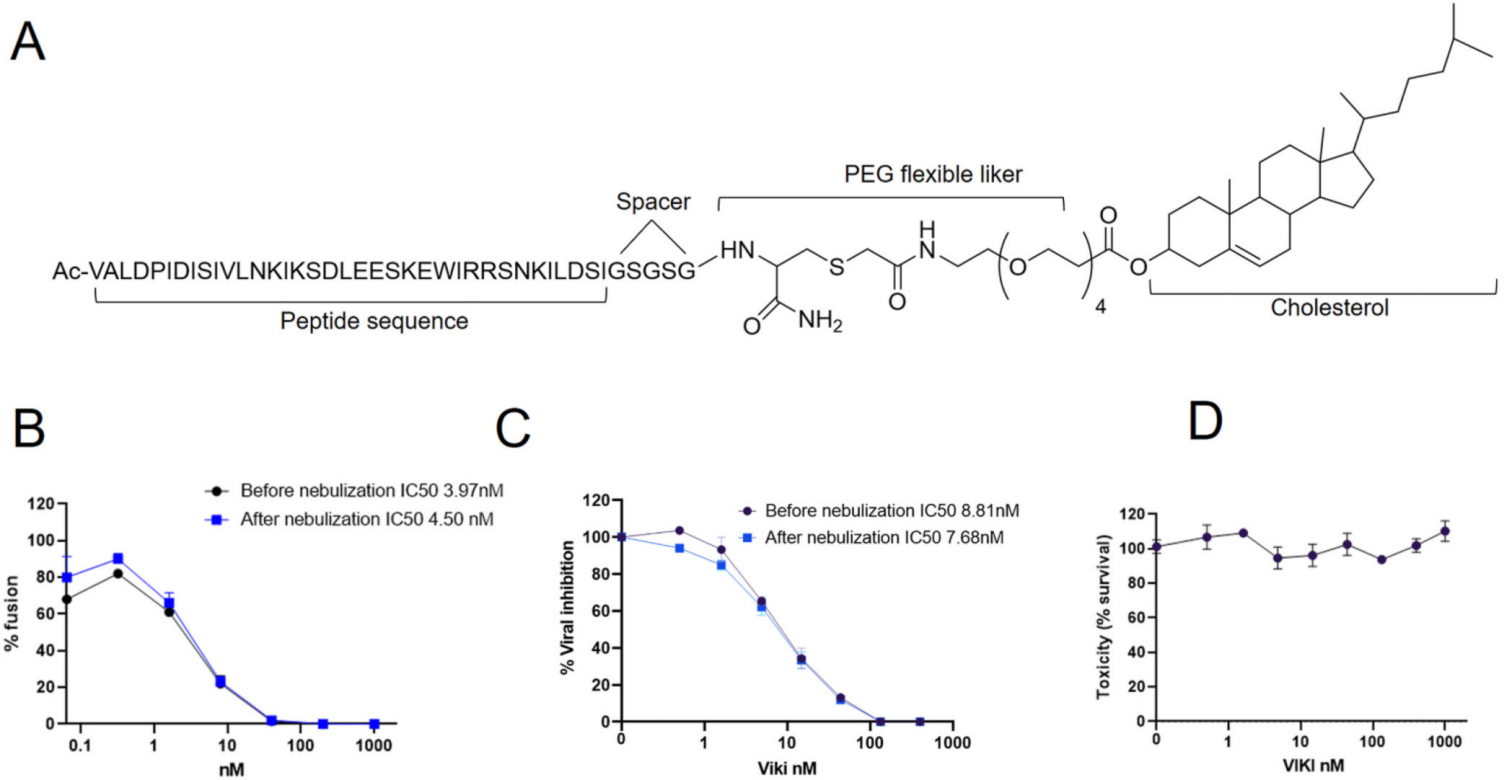
VIKI-PEG4-Chol lipopeptides remain effective after nebulization. (A) Schematic representation of the VIKI lipopeptide; (B) Lipopeptide was nebulized and harvested with an impactor. Serial 3-fold dilutions were performed and analyzed in a fusion assay based on a β-gal complementation assay. (C) Infection inhibition assay based on evaluation of plaque reduction, using 50 PFU of NiV. (D) Cytotoxicity assay was performed by assessing cell viability after 96h in an MTT assay.

The VIKI-lipopeptide used in this study (Fig. 2A) was conjugated to a cholesterol molecule at its C-terminus, as shown previously to improve antiviral properties (Pessi et al., 2012; Porotto et al., 2010a). Both the fusion inhibition assay (Fig. 2B) and dose-response inhibition assay with infectious NiV (Fig. 2C) demonstrated that the activity of the drug was not impaired by its nebulization. Before proceeding to *in vivo* assessment of lipopeptide, we analyzed its potential toxicity *in vitro* using a cell toxicity MTT assay. As shown in Fig. 2D, toxicity was not observed up to 1 μM indicating an *in vitro* Therapeutic Index (Abughazaleh and Tracy, 2007) over 125, meaning that the antiviral dose is over 125 times below the cytotoxic dose (1μM/8 nM).

### 3.3. Biodistribution and safety of VIKI lipopeptide in the AGM model

**Fig. 3.**
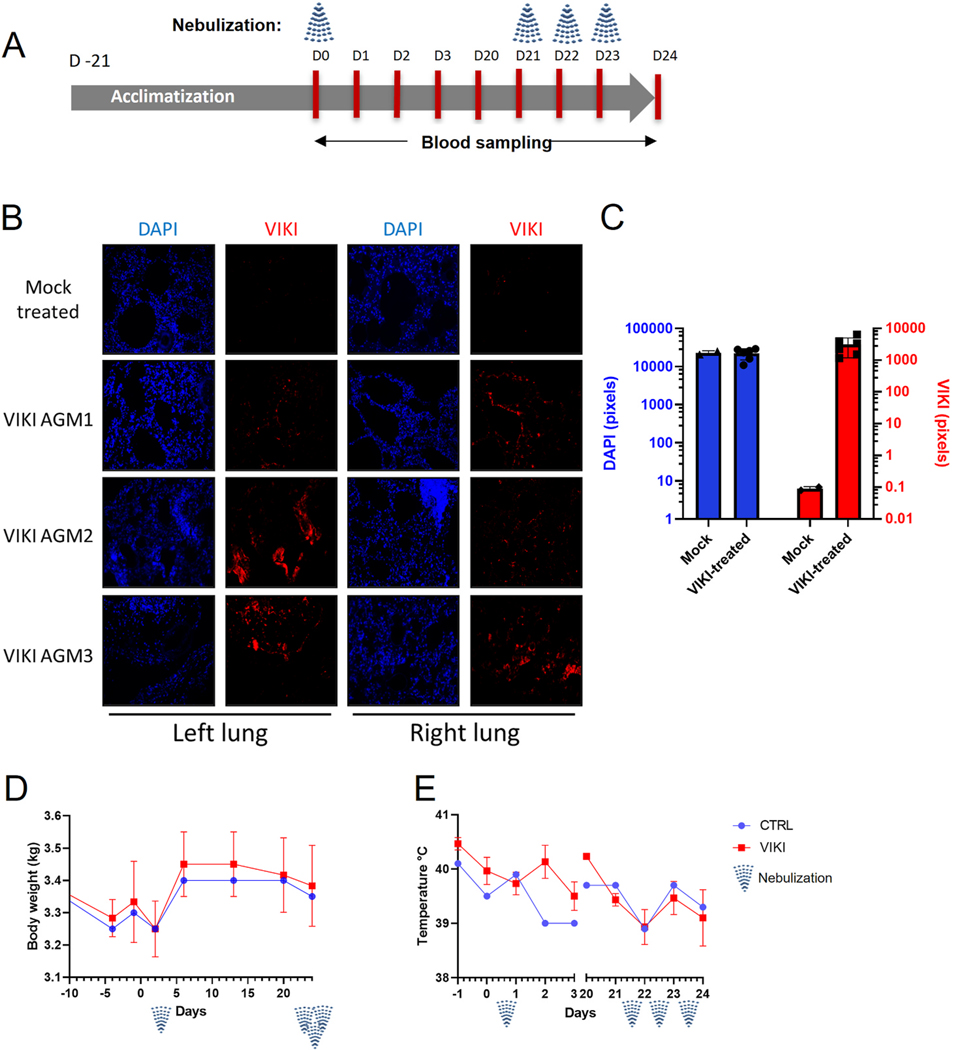
Deposition of lipopeptides in lungs of AGMs following administration via nebulizer with no measurable toxic effect. (A) Schematic representation of the *in vivo* biodistribution/toxicology study. (B–C) Peptide deposition in the lungs of AGMs treated with nebulized peptide under anesthesia 24h before euthanasia. Staining was performed with rabbit anti-VIKI peptide and goat anti-rabbit Alexa 555, and DAPI was used to stain nuclei. Three AGMs were treated with nebulized VIKI peptide and one with saline solution. Fluorescence intensity of DAPI and VIKI stainings were evaluated using ImageJ software. (D–E) Body weight and body temperature after single or triple treatment with 4 mg of nebulized lipopeptide or with saline.

The safety of VIKI peptide administration to AGMs was evaluated in a two-step protocol where animals underwent an initial nebulization and were monitored thereafter for three consecutive days (hematology and blood biochemistry). After a 21-day pause, AGMs that received three consecutive nebulizations at one-day intervals were evaluated one day after for hematology, blood biochemistry and lipopeptide distribution (Fig. 3A). Lungs were subjected to immunofluorescence using anti-VIKI antibodies to detect the presence of lipopeptides. Staining was observed in different sections from both lungs of all treated animals, while no signal was detected in saline-treated animals (Fig. 3B), suggesting that lipopeptide reached different lung regions following the nebulization of all treated monkeys as visualized and quantified (Fig. 3B and C) and confirming the prediction from the demonstrator described in Fig. 1.

Animals were monitored twice daily for the whole duration of the experiment. Neither weight loss nor pyrexia were observed throughout the evaluation (Fig. 3D and E). Both blood biochemistry and hematology remained within normal range in all animals (Supplementary Figs. 1 and 2). Alanine aminotransferase (ALT) and aspartate aminotrasferase (AST) increased in both treated and untreated groups after nebulization, which may be related to the use of anesthetics. Respiratory rate was slightly decreased during nebulization in both saline and lipopeptides-treated groups, suggesting a stress induced effect of the anesthesia and nebulization procedure, probably due to the utilization of masks in animals, as observed previously (Bertrand et al., 2016).

### 3.4. Evaluation of a mesh nebulizer for lipopeptides in a lethal NiV-Bangladesh infection in AGMs

We determined the NiV-Bangladesh dose required to induce a lethal infection by i.t. inoculation. All three doses we assessed 10^2^, 10^4^ and 10^6^ PFU of NiV were 100% lethal, although with a different time course to lethality ranging from 4 to 5 days for animals challenged with the highest dose 10^6^ PFU up to 8–9 days for those which received 10^2^ PFU (Supplementary Fig. 3A). Animals showed a sudden increase in clinical scores due to the onset of a very severe acute respiratory distress (Supplementary Fig. 3B). Blood analysis showed monocytosis, neutrophilia, lymphopenia and thrombocytopenia, although in the 10^6^ PFU group the effect was much less obvious due to the rapid lethality (Supplementary Fig. 3C). The lowest dose of NiV (10^2^ PFU/AGM), which induced 100% lethality in AGMs, was chosen for an aerosolized treatment experiment.

**Fig. 4.**
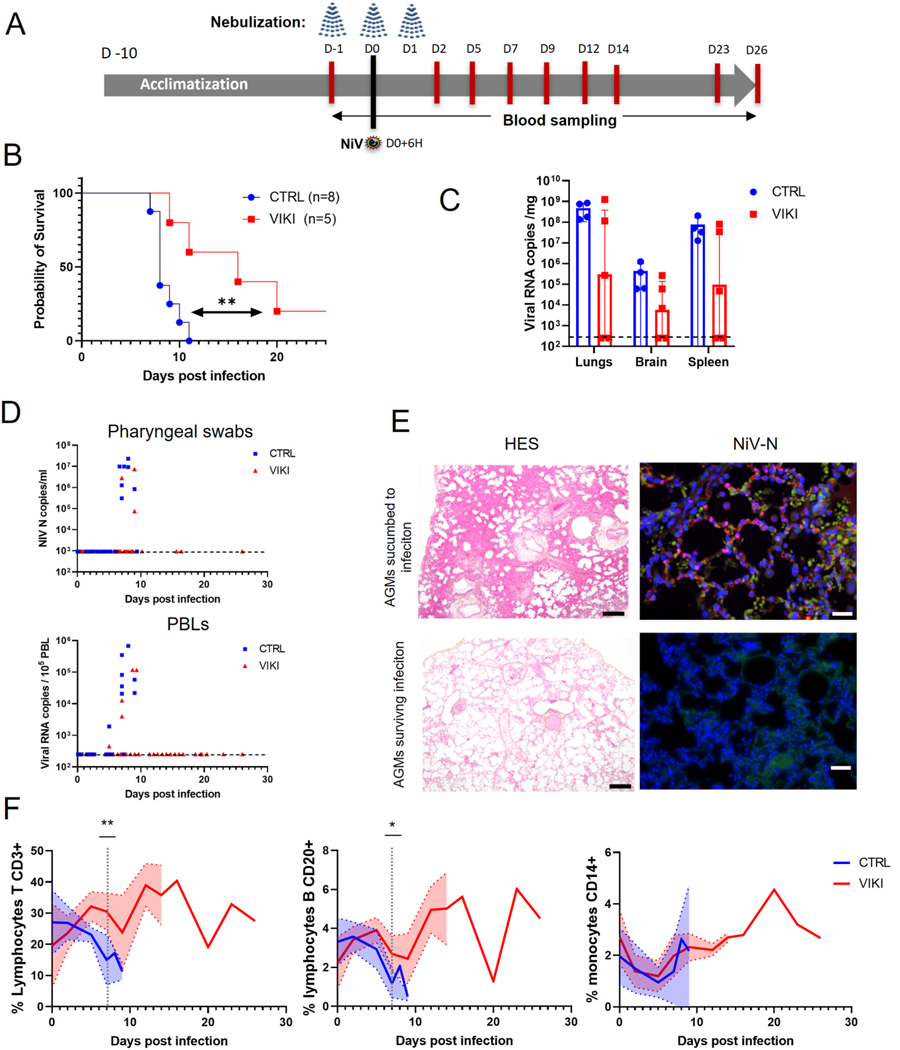
Effect of treatment with nebulized VIKI lipopeptide on NiV-infected AGMs. (A) Experimental design: animals were either treated wtih nebulized mock preparation or with 3 mL of VIKI lipopeptide (4 mg/kg), 24h and 6h before and 24h after intratracheal infection with 100 PFU of NiV. Blood samples were taken on indicateded days (red lines) and animals were followed daily. (B) Kaplan-Meier survival curves of the challenge experiment [VIKI-treated n = 5, and control (CTRL) composed of saline-nebulized animals (n = 3) and untreated historical cohort challenged with 100 PFU, (n = 5)]. Statistical difference analyzed by log-rank Mantel-Cox test. (C) NiV-N RNA quantification by RT-qPCR in animal lung, brain and spleen, obtained after autopsy of control group [n = 2 + 2 historical controls (Pastor et al., 2024)] and treated group (n = 5). (D) Quantification of viral RNA in pharyngeal swabs and Peripheral Blood Leukocytes (PBLs) from AGMs at different time points after infection. (E) Images of representative AGM lung sections from animals surviving or not surviving NiV infection, in haematoxylin-eosin saffron staining (HES, scale bars: 500 μm) and by immunofluorescence using a rabbit anti-NiV N antibody (scale bars: 50 μm). NiV N is marked in red, and nuclei are stained in blue with DAPI. Red blood cells display a green autofluorescence, highlighting the lung architecture. (F) Cytofluorometric analysis of the blood from infected AGMs. CD3^+^ T-lymphocytes, CD20^+^ B-lymphocytes and CD14^+^ monocytes levels were evaluated in blood at time points indicated in 4A and at the time of euthanasia. Results from CTRL group (n = 5) were compared to those from the VIKI group (n = 5). Curves represent the mean of each cell population for each group and the area within filled error bands the standard deviation. Statistical significance was analyzed at 7 dpi, corresponding to the time when all the animals were still alive, and results tested by a *t*-test, followed by a Mann-Whitney test; (*p = 0.05, **p < 0.01).

The protective effect of lipopeptide nebulization was investigated in seven AGMs, with two males receiving a saline solution (CTRL group) and five treated with VIKI lipopeptide (VIKI group). We were unable to conduct a comparison of this with our previous parenteral delivery method that was most successful *in vivo*, due to the limitation of NHP numbers, and therefore cannot compare efficacy with our previous results. All animals received a triple nebulization, 24h and 6h before challenge and 24h after challenge with 10^2^ PFU of NiV and were monitored daily after infection (Fig. 4A). Mock-treated animals (two males and six animals from the previous experiment) developed an acute respiratory distress syndrome and succumbed to infection between days 7–12. Treatment with lipopeptide resulted in significantly prolonged survival (Fig. 4B and Supplemenatry Figs. 4A and 4B) and one animal was alive at the end of the experiment. Another animal was euthanized on day sixteen after developing abnormal tremors on awakening from anesthesia (blood sampling); spleen, lung and brain samples were negative for NiV RNA, suggesting either an anesthetic side effect or an NiV-associated effect. However, viral load analyzed in lungs, spleen and brain by RT-PCR showed a lower average viral level in the peptide-treated AGMs (Fig. 4C) and absence of virus in both the two above animals (the one living at the end of the experiment and the one euthanized for tremors). Virus was not detected in the protected animals neither in pharyngeal swabs nor in PBLs collected during the course of infection (Fig. 4D). In addition, the two AGMs showed neither pathological inflammatory lesions nor NiV-N antigen in lungs, in distinction to animals that developed acute NiV infection and displayed interstitial pneumonia with vasculopathy and the presence of NiV-N antigen in endothelial cells (Fig. 4E), indicating that 40% of AGMs (2 out of 5) were protected from lethal NiV infection. None of the monkeys developed seroneutralizing antibodies.

NiV-infected animals generally had higher C-reactive protein (CRP) and lower albumin levels (corresponding to positive and negative acute phase proteins, respectively), indicating the body’s response to active viral infection, and also high blood urea concentrations, suggesting renal dysfunction. Hematological parameters indicate findings of lymphopenia, monocytosis, neutrophilia and thrombocytopenia that are known signatures of NIV infection in AGMs (Cong et al., 2017; Mire et al., 2019.) (Supplementary Fig. 4,5 and 6). Finally, cytofluorometry analysis of blood cell populations corroborated the previous findings in infected animals (Mathieu et al., 2018; Pastor et al., 2024) as well as in recently reported human patients (Sahay et al., 2024), with a rapid and significant decrease in CD3^+^ T-cells and CD20^+^ B-lymphocytes counts, leading to lymphopenia in untreated control animals. In contrast, in lipopeptide-treated animals these lymphocyte populations were maintained, while classical CD14^+^ monocytes remained similar in both groups (Fig. 4F).

## 4. Discussion

The ongoing outbreaks of NiV in humans and the pandemic potential of henipaviruses highlight the need for surveillance, diagnostics, vaccines, and therapeutics as outlined in the recently-updated henipavirus WHO development priorities roadmap (Anish, 2023; Moore et al., 2024; Spengler et al., 2024). There are currently neither licensed vaccines nor therapeutics for NiV infection. Over the last several decades, we have targeted viral entry for advancing inhibitors of paramyxoviruses and other enveloped viruses that enter cells via the Class I membrane fusion pathway. We have developed an antiviral platform of fusion-inhibitory peptides derived from the HRC domain of paramyxovirus F proteins or from the heptad repeat domains of analogous fusion proteins of other viruses. The approach builds on our finding that lipid conjugation of HRC-derived inhibitory peptides markedly increases antiviral potency and *in vivo* half-life (Pessi et al., 2012; Porotto et al., 2010a). Such peptides effectively inhibit human parainfluenza virus type 3 (HPIV-3), measles virus, influenza virus, SARS-CoV-2, and NiV infection (de Vries et al., 2021; Tiago N. Figueira et al., 2018; T. N. Figueira et al., 2018; Mathieu et al., 2017; Outlaw et al., 2020a; Porotto et al., 2010a).

The fusion-inhibitory peptide platform consists of modular components that can be tuned depending on the virus and disease. For example, peptide dimerization, modification of the lipid conjugate that directs lipopeptide integration into cell membranes, or adjustment of the peptide-lipid linker length, all affect the peptides’ distribution and efficacy for a range of viruses and settings (Figueira et al., 2017; Porotto et al., 2010a). Introduction of non-natural amino acids to decrease the peptides’ protease sensitivity can improve *in vivo* efficacy by prolonging half-life (Outlaw et al., 2019). Peptide amino-acid sequence can also be optimized. In some cases, a fusion-inhibitory peptide based on a specific HRC domain inhibits fusion mediated only by the F protein from which the peptide was derived (e.g., enfuvirtide, an HIV fusion glycoprotein gp41-derived peptide inhibitor is effective against HIV but not against other viruses) (Bolarinwa et al., 2018; Matthews et al., 2004; Rapaport et al., 1995; Wild et al., 1994; Yao and Compans, 1996). Peptides derived from the HRC of HPIV3, however, exhibit activity against not only HPIV3 but also other paramyxoviruses including HeV and NiV (Figueira et al., 2017; C. Mathieu et al., 2015; Mathieu et al., 2017; Porotto et al., 2010a, 2007). The ability of HPIV3 HRC-derived peptides to potently disrupt the fusion machinery of other paramyxoviruses is potentially important in terms of therapy, and forms the basis for the peptides that we have developed and assessed for NiV infection. A key advantage of this peptide platform is that the lipopeptides can be rapidly produced upon identification of an emerging virus that infects via class 1 fusion mechanism, and readily administered either parenterally or via the respiratory route, providing options for delivery depending on the specific viral infection.

The lipopeptide used in the current study, “VIKI-PEG4-Cholesterol”, which inhibits both HPIV3, HeV, and NiV, was previously assessed *in vitro*, ex vivo and *in vivo* in several rounds of optimization (Augusto et al., 2017; Mathieu et al., 2017; Pessi et al., 2012; Porotto et al., 2006, 2007, 2009, 2010b). Intraperitoneal treatment of Syrian hamsters 2 days before challenge with NiV-Malaysia provided 100% protection against lethal NiV infection for at least 21 days after exposure to the lethal inoculum. Treatment concurrent with infection led to an 80% survival rate and surprisingly given the rapid progression of NiV disease, even treatment 2 days after infection led to survival of 40% of the animals (Porotto et al., 2010a). In an effort to assess the respiratory route for peptide delivery, a route that has led to effective HRC-peptide treatment for measles virus and SARS-CoV-2 (de Vries et al., 2021; Reynard et al., 2022; Mougari et al., 2025), we previously used a peptide similar to the current lipopeptide but conjugated with tocopherol (“VIKI-PEG4-Tocopherol”) delivered intranasally in hamsters and intratracheally in AGM (Mathieu et al., 2018). Intranasal peptide dosing of Syrian hamsters infected with high dose (10^6^ PFU) of NiV Malaysia for 3 days at the start of infection resulted in 50% survival suggesting that peptide delivery through the respiratory route alone was inadequate to fully prevent infection. Intratracheal peptide dosing in NHPs infected with a very high dose of NiV Malaysia (2 × 10^7^ pfu) was affected by expectoration of the peptide on the first day of treatment in most treated animals (Mathieu et al., 2018) hindering definitive assessment of the effectiveness of airway delivery in NHPs. Taken together, despite the difficulties in interpretation, it seems likely that the airway route of delivery alone is not sufficient to fully inhibit NiV infection *in vivo* and would form part of a treatment strategy. These delivery route challenges prompted our effort to develop an aerosolized delivery method for NiV treatment, aimed at overcoming the limitations initially encountered with respiratory tract delivery, to evaluate the potential of airway delivery as a viable component of antiviral therapy. Such a route would also be clinically applicable for lipopeptide delivery to the airway.

We engineered and refined an aerosol delivery system for AGMs that combines a mesh nebulizer with a face mask, using an artificial respiratory model. This delivery approach was tested in an AGM model for NiV infection to showcase the nebulizer’s capabilities and assess the safety of lipopeptide nebulization in NHPs. Exposure to nebulized peptide elicited no side effects in the AGMs. Additionally, a treatment trial was conducted where aerosolized doses of VIKI-PEG4-Chol were delivered using the nebulizer device one day before infection, on the day of infection, and one day after exposure to the highly lethal NiV-Bangladesh. Administering only nebulized peptide, without supplementary delivery routes, with treatment ending just one day post-infection, still resulted in 40% efficacy and significantly delayed mortality. Unfortunately, study limitations prevented a direct comparison between different delivery routes, or the evaluation of a combined approach, which we anticipate may be essential for fully inhibiting NiV infection. While the aerosol delivery route of antiviral lipopeptides provided full protection of macaques against measles virus infection (Reynard et al., 2022), a combined airway and systemic delivery may be required to inhibit NiV.

We anticipate that this nebulizer-based aerosol delivery system, optimized for AGMs, will be a valuable tool for NiV antiviral research. While AGMs are considered the most accurate model for NiV infection in humans, differences in breathing patterns between AGMs and humans must be accounted for when translating these findings to human trials (MacLoughlin et al., 2016; Creppy et al., 2024). Future studies in AGMs will explore the comparative efficacy of different delivery routes and their combinations. Additionally, next-generation versions of the anti-HPIV3/NiV fusion inhibitory peptide and peptides incorporating protease resistance modifications to improve peptide stability (Outlaw et al., 2020b, 2020c, 2021) will be tested. These efforts aim to pave the way for the development of an aerosol delivery method to support rapid antiviral responses against airborne viruses.

## Supplementary Material

Supp

Appendix A. Supplementary data

Supplementary data to this article can be found online at https://doi.org/10.1016/j.antiviral.2025.106095.

## Data Availability

Data will be made available on request.
