# Supplementary material for: Development of nebulized inhalation delivery for fusion-inhibitory lipopeptides to protect non-human primates against Nipah-Bangladesh infection
Source: Antiviral Res. Author manuscript; Available in PMC 2025 Jul 1. (PMC12212376; doi:10.1016/j.antiviral.2025.106095)
Supplement: Supp [file NIHMS2082847-supplement-Supp.pdf]

Protection of non-human primates from respiratory Nipah virus infection by nebulized fusion-inhibitory lipopeptide

Supplementary figures:

Supplementary Figure 1. Effect of lipopeptide nebulization on the biochemical parameters in blood and breathing rate in AGMs

Supplementary Figure 2. Effect of lipopeptide nebulization on the evolution of haematology parameters in AGMs

Supplementary Figure 3. Characterization of NiV infection of AGMs following intra-tracheal route of viral administration

Supplementary Figure 4. Evolution of the biochemical parameters in blood of AGMs infected with different NiV doses

Supplementary Figure 5. Survival curves and blood biochemistry analysis of AGMs involved in lipopeptide nebulization study

Supplementary Figure 6. Evolution of the blood haematology during the lipopeptide-treated AGM NiV-challenge study.

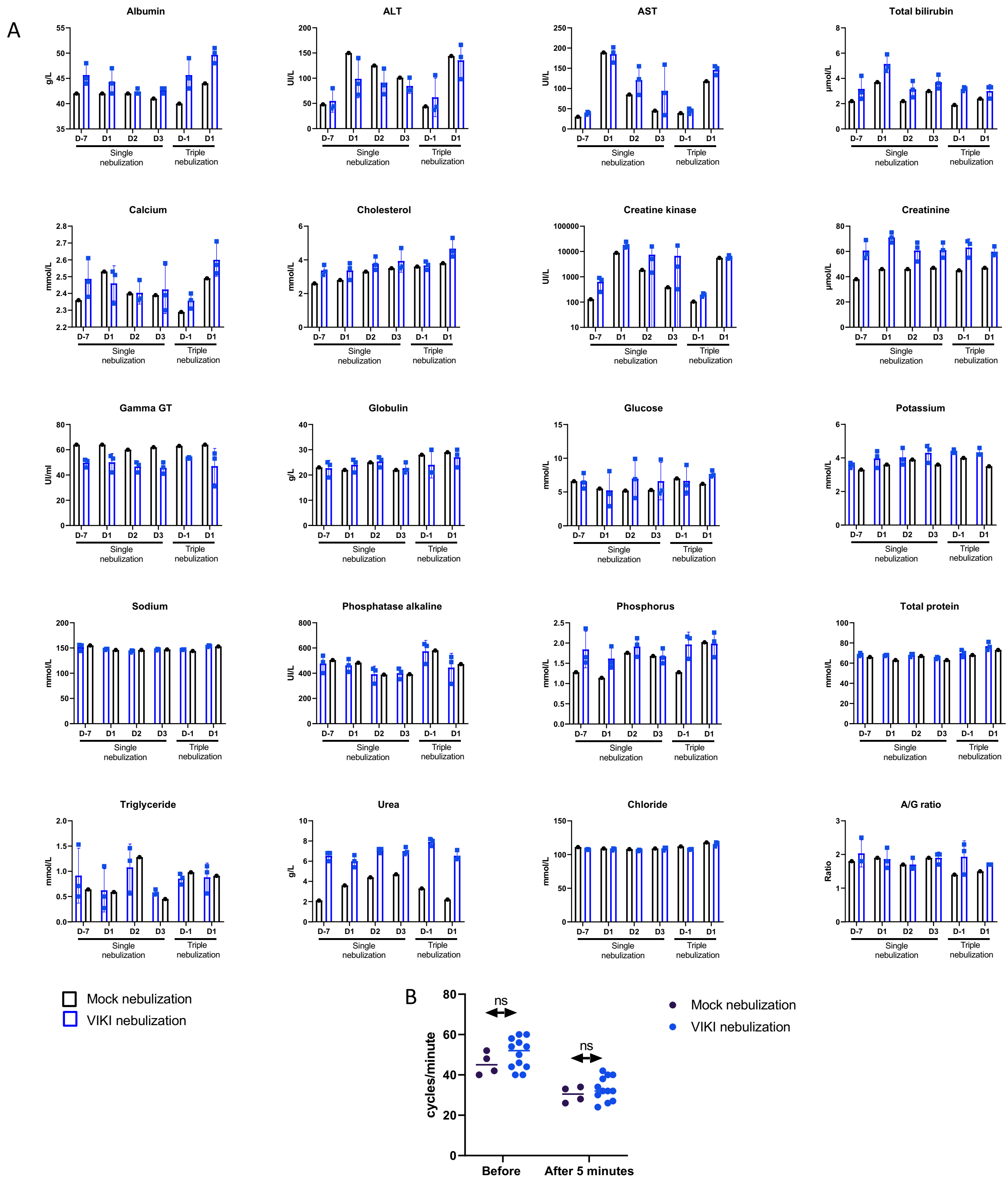

Supplementary Figure 1. Effect of lipopeptide nebulization on the biochemical parameters in blood and breathing rate in AGMs. Follow-up of blood biochemistry (A) and breathing rate (B) during the *in vivo* safety study in AGMs. Blood biochemistry was analysed on a KONELAB KL30 ISEND (Thermo). Breathing rate was visually recorded by the veterinarian. ALAT, Alanine aminotransferase; ASAT aspartate aminotrasferase.

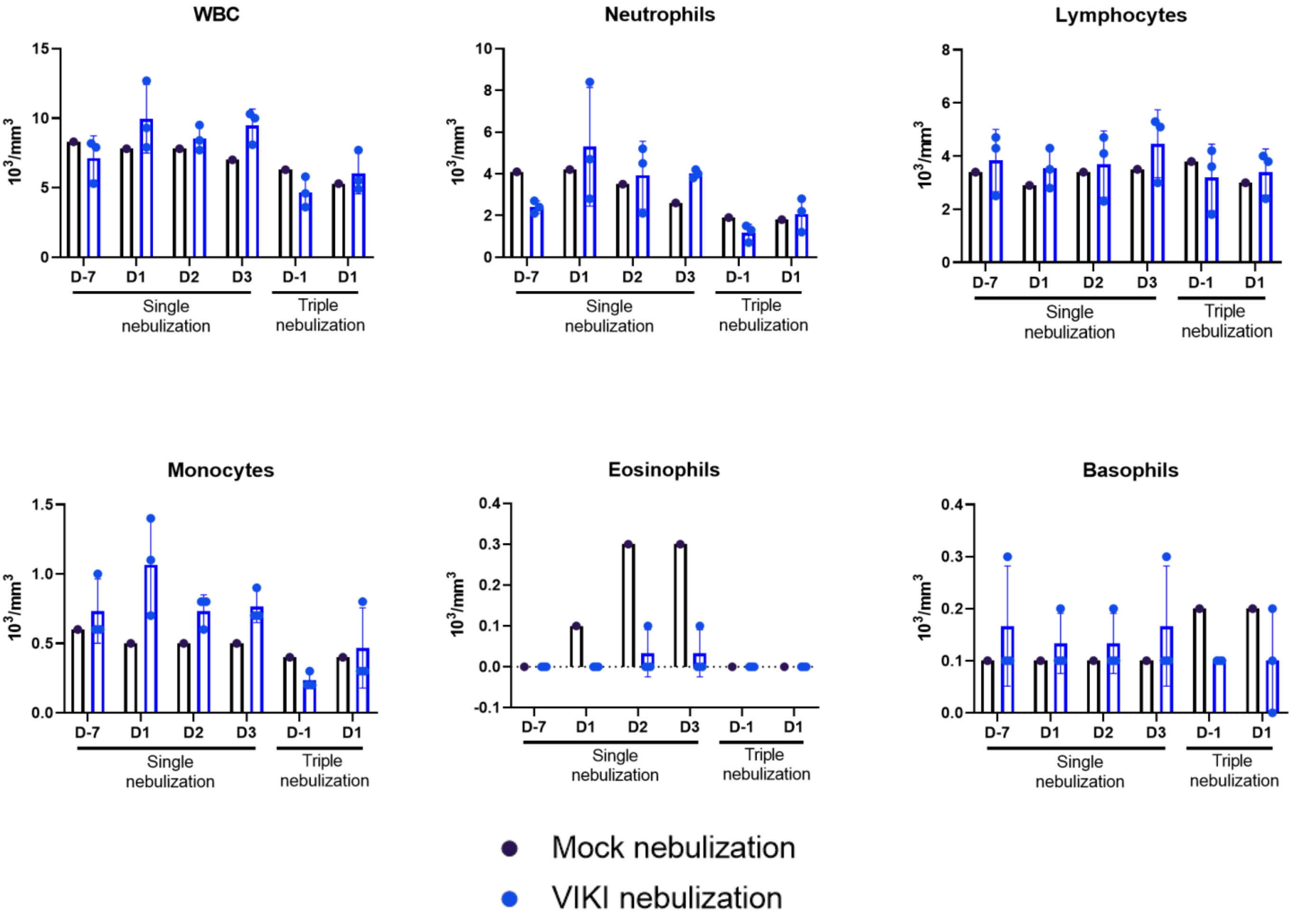

Supplementary Figure 2. Effect of lipopeptide nebulization on the evolution of haematology parameters in AGMs. Follow-up of blood haematology during the in vivo safety study. Blood haematology was analysed on a XT2000i Vet automatic analyser (Sysmex). WBC: white blood count.

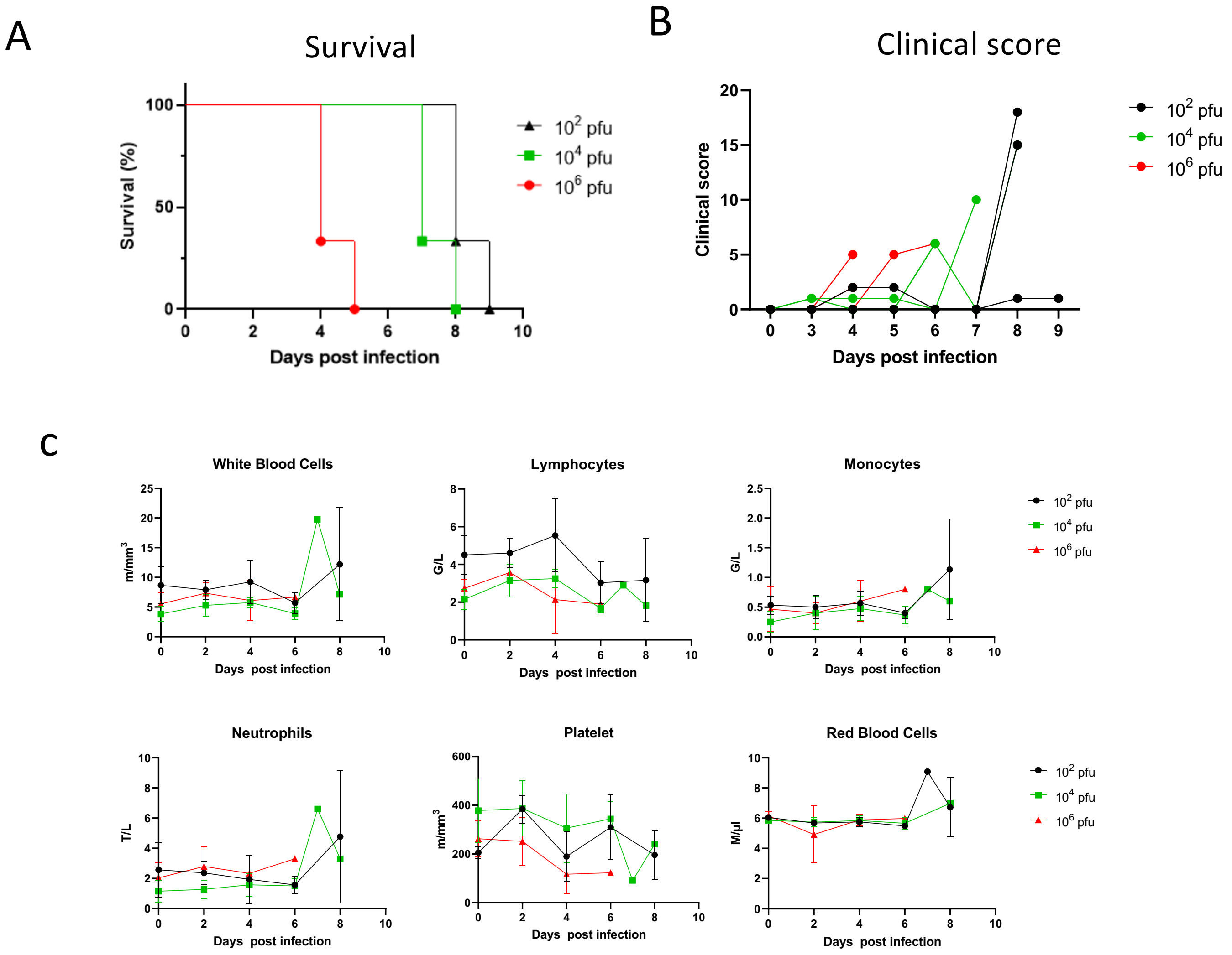

Supplementary Figure 3. Characterization of NiV infection of AGMs following intra-tracheal route of viral administration. Ten AGMs were challenged intra-tracheally with NiV and monitored during 10 days. Three groups were composed that received 10^2 PFU (n=3♀), 10^4 PFU (n=2♀+2♂), and 10^6 PFU (n=3♂) in a 2ml inoculum. A. Kaplan-Meier survival curve. B. Daily monitoring of animal's clinical score. C. Blood haematology analysed every two days on a MS9 analyzer, MS Labo.

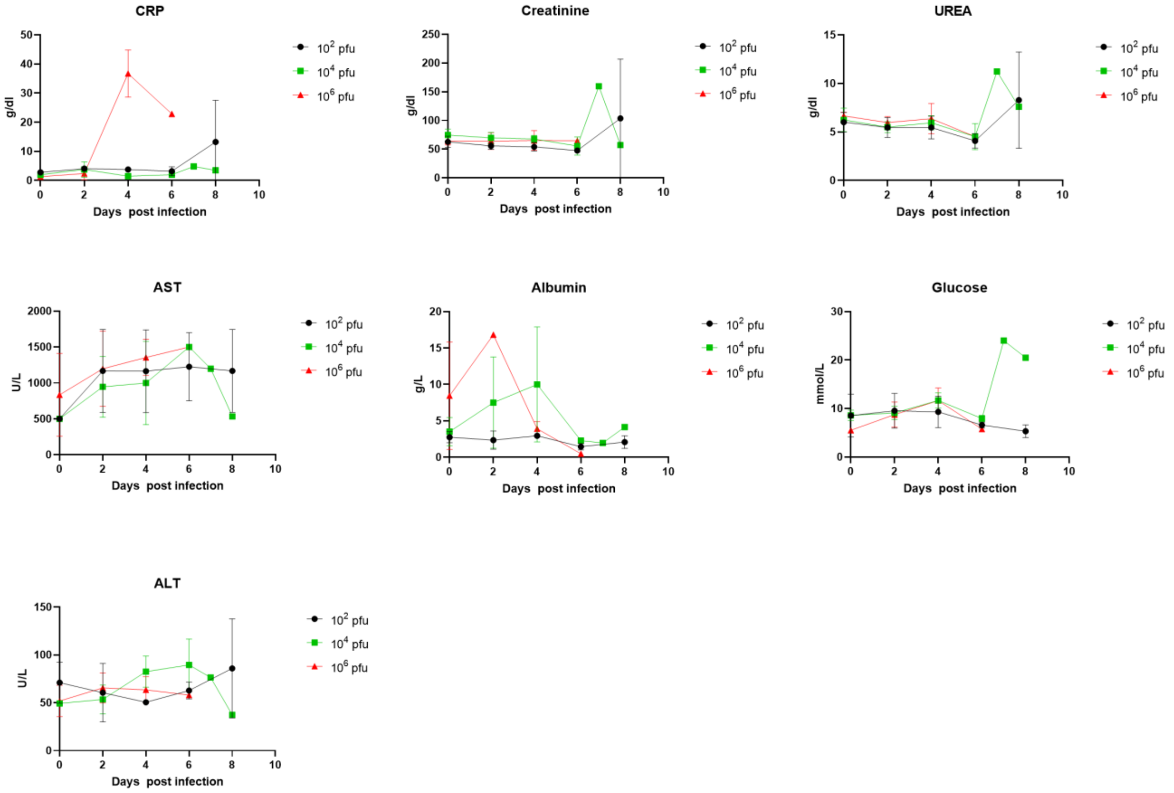

Supplementary Figure 4. Evolution of the biochemical parameters in blood of AGMs infected with different NiV doses. Blood biochemistry was analysed every two days on a Pentra C200 analyser, Horiba.

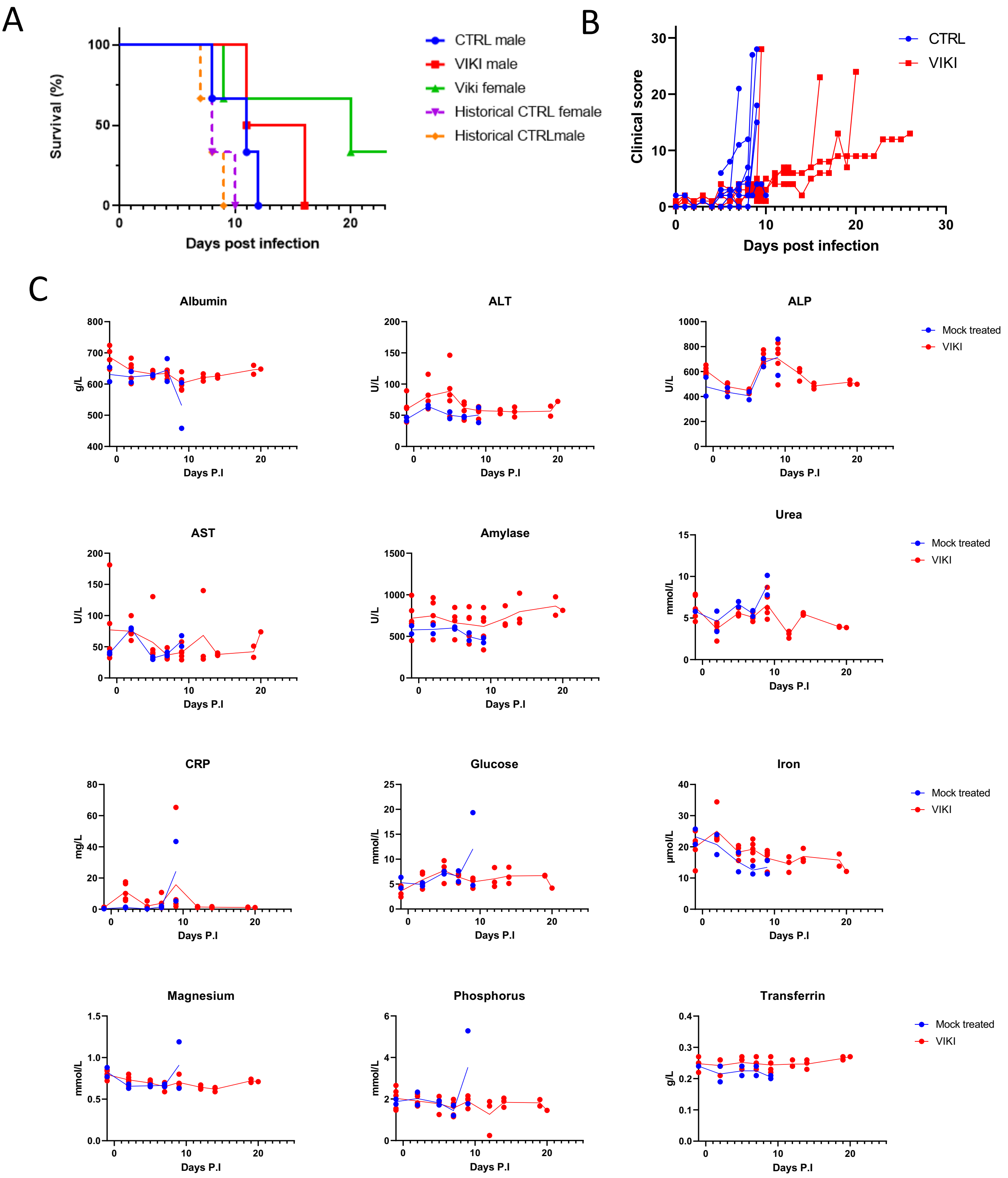

Supplementary Figure 5. Survival curves and blood biochemistry analysis of AGMs involved in lipopeptide nebulization study. A. Detailed Kaplan-Meier survival curve. B. C. Clinical score of the animals shown in 5B. C. Blood biochemistry analysis of nebulized animals. CRP C reactive protein.

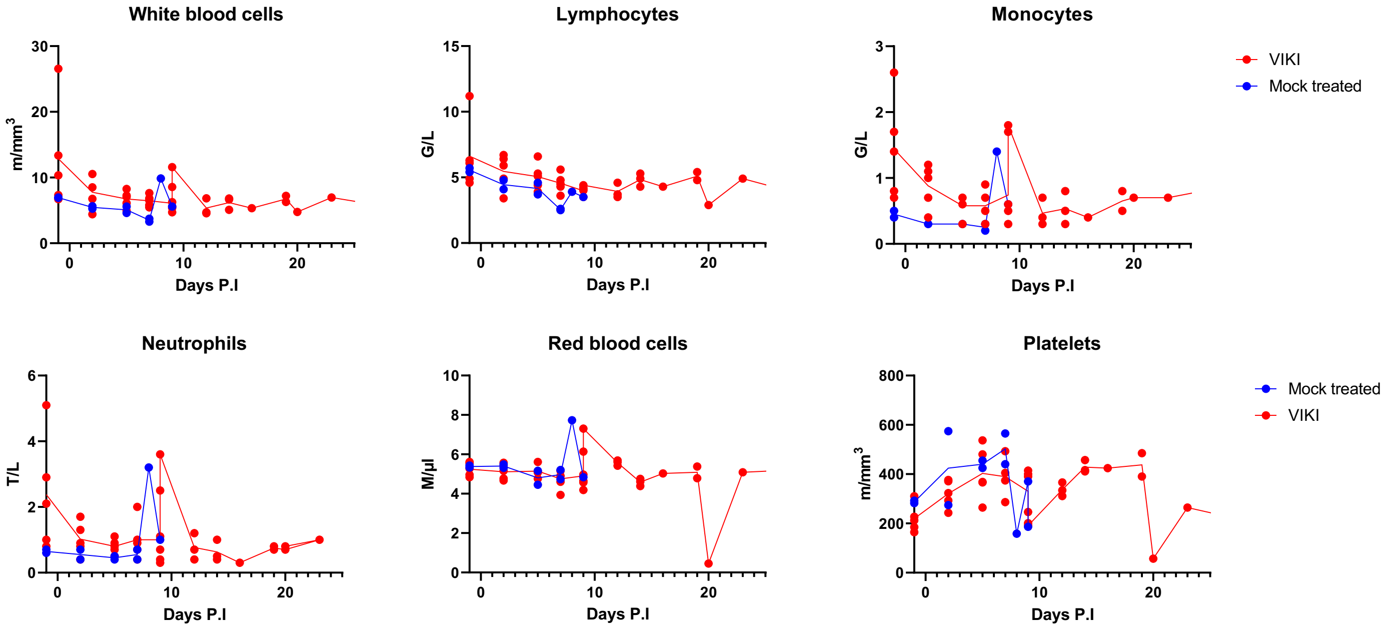

Supplementary Figure 6. Evolution of the blood haematology during the lipopeptide-treated AGM NiV-challenge study. Follow-up of blood haematology every two days on a MS9 analyser, MS Labo.
